# Multiview clustering of multi-omics data integration by using a penalty model

**DOI:** 10.1186/s12859-022-04826-4

**Published:** 2022-07-21

**Authors:** Hamas A. AL-kuhali, Ma Shan, Mohanned Abduljabbar Hael, Eman A. Al-Hada, Shamsan A. Al-Murisi, Ahmed A. Al-kuhali, Ammar A. Q. Aldaifl, Mohammed Elmustafa Amin

**Affiliations:** 1 School of Mathematics and Statistics, Lanzhou University, Lanzhou, China; 2 Department of Mathematics, Faculty of Science and Technology, Omdurman Islamic University, Khartoum, Sudan; 3 Department of Data Science and Information Technology, Taiz University, Taiz, Yemen; 4 School of Management, Lanzhou University, Lanzhou, China; 5 School of Information Engineering, Wuhan University of Technology, Wuhan, China

**Keywords:** Multi-omics data, Multiview clustering, Data integration, Penalty model

## Abstract

**Background:**

Methods for the multiview clustering and integration of multi-omics data have been developed recently to solve problems caused by data noise or limited sample size and to integrate multi-omics data with consistent (common) and differential cluster patterns. However, the integration of such data still suffers from limited performance and low accuracy.

**Results:**

In this study, a computational framework for the multiview clustering method based on the penalty model is presented to overcome the challenges of low accuracy and limited performance in the case of integrating multi-omics data with consistent (common) and differential cluster patterns. The performance of the proposed method was evaluated on synthetic data and four real multi-omics data and then compared with approaches presented in the literature under different scenarios. Result implies that our method exhibits competitive performance compared with recently developed techniques when the underlying clusters are consistent with synthetic data. In the case of the differential clusters, the proposed method also presents an enhanced performance. In addition, with regards to real omics data, the developed method exhibits better performance, demonstrating its ability to provide more detailed information within each data type and working better to integrate multi-omics data with consistent (common) and differential cluster patterns. This study shows that the proposed method offers more significant differences in survival times across all types of cancer.

**Conclusions:**

A new multiview clustering method is proposed in this study based on synthetic and real data. This method performs better than other techniques previously presented in the literature in terms of integrating multi-omics data with consistent and differential cluster patterns and determining the significance of difference in survival times.

## Background

Cancer is a complicated disease with phenotypic manifestations connected to various molecular signatures, such as gene expression and DNA methylation. That is, genetic mutations and epigenetic landscapes can be used to determine cancer types and subtypes. Consequently, any causal analysis based exclusively on one component or single omics will exhibit a causal reductionism resulting in unsatisfactory results [1]. The rapid advancement of high-production technology has produced a massive amount of data generated from patients with different types of cancer, facilitating the collection of different genome-scale datasets to address clinical and scientific challenges. The Cancer Genome Atlas (TCGA) is one of the most prominent projects. It provides a considerable amount of omics data obtained from different platforms (e.g. DNA methylation and gene expression). Therefore, developing methods for integrating different types of omics data and creating a comprehensive picture of a given disease or biological process is necessary to improve disease detection, treatment and prevention [2].

Clustering is a popular technique for exploratory data analysis [3], and it has been used as a fundamental step in the comprehensive analysis of omics data to identify cancer subtypes [4–6] and detect correlated gene expression patterns [7]. Traditional clustering methods for omics data analysis involve one data type, such as DNA methylation [8]. However, information from one data type may be inconsistent due to a small number of samples compared with a large number of measurements, scale differences, collection bias, noise in each dataset and the complementary nature of information provided by different types of omics data. Several methods have focused on multi-omics data clustering to integrate information from different types of omics data [9–12] or used concordant data structure to perform clustering [10, 12–16].

Although multiview clustering methods have significantly improved clustering performance, some methods assume that underlying clusters for different data types are consistent clusters [11, 12, 16], whilst others assume that such clusters are differential clusters [17–19]. In such case, multi-omics data should have simultaneous consistent and differential cluster patterns in accordance with our intuition. A few integrative clustering methods have been proposed recently to integrate multiview data with consistent and differential cluster patterns [20–23]. The authors of [24] proposed a multiview clustering method to solve the multiview spectral clustering optimisation problem [17]; this method used the linear search technique. However, all previous studies reported low performance in integrating different data types. The optimisation problem exhibits orthogonality constraints. Several methods have been proposed to solve the optimisation problem with orthogonality, such as gradient-based methods [24–26], conjugate gradient methods [27, 28], projection-based methods [29], a constraint-preserving updating scheme [30, 31], a multiplier correction framework [32] and penalty function methods [33].

In the present study, we proposed a new approach for multiview clustering that aimed to integrate different types of data with consistent and differential cluster patterns. The current work developed the computational framework for multiview clustering presented in [23] to improve performance in integrating different types of omics data with consistent and differential cluster patterns. The procedures of the proposed method were based on the penalty model, and the first-order algorithm in [33] was used to solve the multiview spectral clustering optimisation problem with orthogonality constraints [21]. Moreover, we evaluated the performance of the proposed method on synthetic data. On the basis of the obtained high accuracy, we used the method on four different types of real multi-omics data. The experiment results demonstrated that the proposed method outperformed other methods presented in the literature.

The major contributions of this work are as follows. Firstly, a new multiview clustering method is provided for integrating multi-omics data with consistent and differential cluster patterns. Secondly, the proposed method is used to determine the significance of difference in survival times. Finally, comprehensive experiments on several state-of-the-art multiview clustering methods are performed to validate the proposed method. The remaining sections of this paper are organised as follows. "Related work" section provides a review of related work. "Proposed method" section presents the proposed method. "Results and discussion" section describes the experiments performed on datasets by using state-of-the-art algorithms for comparison. "Conclusion" section concludes the study.

## Related work

In this section, we review related work on multiview clustering methods for integrating different types of data.

Currently available approaches can be classified into three types. The first type is based on a concordant data structure when performing clustering [10, 12–16]. iCluster is an integrative clustering method based on a Gaussian latent variable model with lasso-type penalty terms to induce sparsity in coefficient matrices for feature selection. This approach’s significant computational complexity necessitates gene preselection. Therefore, clustering outcomes are dependent on this step [12, 15]. To address the issue of gene preselection, a fused network method is proposed. This method uses sample networks as a foundation for integration and merges similarity networks built for each data type into a single combined similarity network via an iterative approach based on message passing. The final clusters for a fused network are obtained using spectral clustering [13]. A multiple kernel learning method that aims to reduce the dimensions of data from different sources conserves the distance of neighbours for all data types by extending spectral clustering to accept several affinity matrices as input and then fuses matrices by using a linear combination with weight optimisation [12].

The second type is based on spectral clustering. The affinity aggregation spectral clustering method provides a framework for learning the spectral clustering similarity matrix to improve the robustness of spectral clustering by reducing the effect of unreliable and irrelevant features [34]. The authors of [21] and [22]aimed to maximise consistency between clusters from different perspectives by using various cluster consistency measures. The formulation of an optimisation problem in [22] included an alternative computation of the eigenvectors of the regularised Laplacian matrix; thus, the method is less stable and more likely to yield the local optimum.

For the third type, multiview clustering methods have demonstrated remarkably improved clustering performance, although some of these methods assume that the underlying clusters across different data types are consistent clusters. For example, methods based on the application of cancer patients assume that the underlying subtypes are the same across different data types [11, 12, 16]. The flaw of these methods is that they do not check whether genes, microRNA (miRNA) and other small molecules exhibit the same cluster patterns across different subtypes. Several methods based on the assumption that underlying clusters across various data types are different have also been proposed by comparing clusters identified in different data or by merging information that demonstrates differences in single objects [17–19]. Meanwhile, a few methods have focused on the problem of integrating different datasets with consistent and differential cluster patterns. The method in [22] tends to obtain the local optimum, whereas the methods in [21, 35] overly relax the original multiview ratio cut model, likely producing the information in each data type. The multiview clustering method based on manifold was proposed to solve the multiview spectral clustering optimisation problem [23] by using a linear search technique. All these methods exhibit lower performance in integrating different data types. Therefore, we proposed a new multiview clustering method to obtain better performance.

## Proposed method

In this section, we introduce the penalty model, the proposed method steps, the algorithm, and the example for our method as follows.

### Penalty model

By considering the following matrix optimization problem with orthogonality constraints:

1 $$\begin{aligned} \min _{X \in R^{n \times p}} f(X), s.t. X^{T}X=I_{p}, \end{aligned}$$

where $I_{ p}$ is any $p \times p$ identity matrix, and $f: R^{n \times p}\mapsto R$ satisfies the following assumption throughout this section. We start with the merit function that can be written as follows:

2 $$\begin{aligned} h(X)=f(X)-\frac{1}{2}\left\langle \psi (\nabla f(X)^{T}X,X^{T}X-I_{p})\right\rangle +\frac{\beta }{4}\begin{Vmatrix} X^{T}X-I_{p} \end{Vmatrix}_{F}^{2} \end{aligned}$$

This merit function was firstly defined in Gao et al. [36]. Equation (4.2) evaluates the function value reduction of the proximal linearised augmented Lagrangian algorithm (PLAM), where $\psi :R^{p \times p}\rightarrow R^{p \times p}$, and $\psi (X)= \frac{X^{T}+X}{2}$ denotes the linear operator for symmetrisation. The penalty model that minimises *h*(*X*) under a compact convex constraint (PenC) is as follows:

3 $$\begin{aligned} \min _{X\in M } h(X), \end{aligned}$$

where *M* is a compact convex set that contains $St_{n,p}$.

Xiao et al. [33] demonstrated that the original problem 1 and the penalty model 3 are equivalent. They proposed the first-order method (PenCF) for solving problem 3. In our work, we used PenCF to solve our problem because our objective function was formulated as

4 $$\begin{aligned} f\left( X\right) = \ Tr\left( X^{T} L X \right) . \end{aligned}$$

### Steps of the proposed method

The proposed method has five steps. Firstly, to normalise each data type to have a standard normal distribution [10], we convert each data type into a patient–patient k-nearest neighbour (NN) similarity network on the basis of a spectral clustering method [37, 38]. Secondly, we obtain a multiview spectral clustering optimisation problem from the multiview network clustering model to integrate multiple similarity networks [21, 35]. Thirdly, we use the penalty model, i.e. the first-order algorithm from [33], to solve the multiview spectral clustering problem with orthogonality constraints. Fourthly, we repeat the processing of the penalty model until convergence is achieved, and we obtain the values of *X* that are represented by the matrix that contains the label for each patient. Finally, we use *k* -mean to cluster the matrix *X* into ${C_{1};C_{2};C_{3};...;C_{K}}$. We present the details of these steps as follows.

#### Normalization

We start to normalise each feature across all data types to have a standard normal distribution. We use the following form:

5 $$\begin{aligned} {{\check{g}}=\frac{g-E(g)}{\sqrt{Var(g)}}}, \end{aligned}$$

where ${\check{g}}$ denotes the feature after normalization, *g* denotes a feature of any data type, *E*(*g*) represents the mean of features, and *Var*(*g*) represents the variance of features.

#### Construction of the k-NN similarity network

Consider *M* types of omics data measurements $\left\{ X_{m} \right\} _{m=1}^{M}$ (each with a dimension of ${\check{g}}_{m}$ ) collected from *N* patient samples, such that $X_{m}$ is a ${\check{g}}_{m} \times N$ matrix, where ${\check{g}}_{m}$ is the number of features of *m*. For each data type $X_{m}$, we construct a patient-to patient similarity network $G_{m}$ to model the local neighbourhood relationships between the samples. Let $G_{m} = \left( V_{ m}; E_{m}; A_{ m} \right)$ denote a patient similarity network for data type *m*, where $V_{ m}=\left\{ {v_{1};v_{2};v_{3}...;v_{N}} \right\}$ denotes the set of the nodes, $E_{m}$ denotes the edge set, and $A _{m}$ denotes the adjacency matrix. The nodes represent *N* patients, and the edges represent the connection between patients.

The adjacency matrix $A_{m}$ of the network $G _{M}$ is is a symmetric matrix whose entry $a_{m} (i,j)$ represents the edge weight if there is an edge between node $v_{i}$ and $v_{j}$, otherwise, $A_{m} (i, j) = 0$. To construct this similarity network, we firstly compute a similarity matrix for measuring pairwise similarity between each sample pair. Here, we use a common similarity measure, called Gaussian similarity, as the similarity metric [37, 38]:

6 $$\begin{aligned} {{{\textbf {S}}}_{m}(i,j)=\exp \left( \dfrac{d^{2}}{2\alpha ^{2}} \right) } \, i=1,2,\cdots ,N, j=1,2,\cdots ,N; \end{aligned}$$

where $d=||x_{m}(i)-x_{m}(j)||$ is the Euclidean distance between $x_{m}(i)$ and $x_{m}(j)$ patients, and parameter $\alpha$ controls the width of the neighbourhoods. Several options are available for setting parameter $\alpha$, here we use the most common choice by setting parameter $\alpha$ as the standard deviation of patients $||x_{m}(i)-x_{m}(j)||$. Then, we construct a *k*-NN network from the similarity matrix ${{\textbf {S}}}_{m}$. We symbolise ${{\textbf {N}}} _{ i}$ as a set of node $v _{i}$ neighbours with node $v _{i}$, and the size of ${{\textbf {N}}} _{ i}$ is equal to *k*. We then connect $v _{i}$ and $v _{j}$ with an undirected edge with the edge weight as ${{\textbf {S}}} (i,j)$ if $v _{i}\in {{\textbf {N}}} _{ i}$, as shown in Eq. (7).

7 $$\begin{aligned} A_{m} (i, j)= {\left\{ \begin{array}{ll} {{\textbf {S}}}_{m}(i,j),&{}\quad \text {if }v_{i} \in {{\textbf {N}}} _{ i} \\ 0,&{}\quad \text {otherwise}. \end{array}\right. } \end{aligned}$$

Thus far, the *k*-NN similarity network $G_{m}$ for each data type is constructed.

#### Multiview network clustering model for integrating the multiple similarity networks

To integrate all *k*-NN similarity networks, we first compute the Laplacian matrix $L_{m}$ for each *k*-NN similarity network $G_{m}$ for all data types.

8 $$\begin{aligned} {L_{m}=D_{m} -A_{m}}, \end{aligned}$$

where $A_{m}$ is the adjacency matrix for the *k*-NN similarity network $G_{m}$; and $D_{m}$ is the diagonal matrix for the *k*-NN similarity network $G_{m}$, whose entries are the degree of all the nodes(patients) in the *m*-th $G_{m}$.

Then, we present the multiview network clustering model [21, 35]. We start by denoting the number of clusters as *K* across the *M* networks and $S^{m}$ as the assignment of the *N* nodes into *K* clusters for the network $G_{M}$.

We set

9 $$\begin{aligned} S_{{i,k}}^{m} = \left\{ {\begin{array}{*{20}l} {1,} \hfill & {{\text{if}}\;i \in G^{m} \;belongs\;to\;the\;k - th\,cluster} \hfill \\ {0,} \hfill & {{\text{otherwise}}.} \hfill \\ \end{array} } \right. \\ \quad i = 1;2; \ldots ;N,m = 1;2; \ldots ;M,k = 1;2; \ldots ;K. \\ \end{aligned}$$

We firstly align the clusters obtained for different networks to show consistent and differential cluster patterns across multiple networks. The computation is complex if we directly align the identified cluster patterns in each network. Thus, Zhang et al. proposed identifying cluster patterns for each network and aligning clusters for multiple networks. The cluster patterns in each network are identified using spectral clustering. For cluster alignment in different networks, the similarity between the *m*th cluster in network $G_{m}$ and the *m*th cluster in network $G_{h}$ is defined as $\dfrac{ S_{.,k}^m S_{.,k}^h}{||S_{.,k}^m|| _{2} ||S_{.,k}^h|| _{2} }$. Zhang et al. [21] and Chen et al. [35] aimed to maximize the similarities of the corresponding clusters in all networks, and the objective function of a multiview network clustering model is

10 $$\begin{aligned} \varphi \left( S^{1},S^{2},..,S^{M}\right) = \sum _{m=1}^{M} \sum _{k=1}^{K}\dfrac{\left( S_{.,k}^m\right) ^T \left( D_{m}-A_{m}\right) S_{.,k}^m }{\left( S_{.,k}^m\right) ^T S_{.,k}^m } -\beta \sum _{m,h=1}^{M} \sum _{k=1}^{K}\dfrac{\left( S_{.,k}^m\right) ^T S_{.,k}^h}{||S_{.,k}^m|| _{2} ||S_{.,k}^h|| _{2} }, \end{aligned}$$

where $\beta$ is the parameter for controlling the contributions from intra- and inter-network connections. The optimization problem is formulated as

11 $$\begin{aligned} & \min \varphi \left( {S^{1} ,S^{2} , \ldots ,S^{M} } \right) \hfill \\ &s.t.\quad S_{{i,k}}^{m} \in \left\{ {0,1} \right\},\;i = 1,2,3, \ldots N;\;m = 1,2,3, \ldots M;\;k = 1,2,3, \ldots K \hfill \\ &\sum\limits_{{k = 1}}^{K} {S_{{,k}}^{m} } = 1\quad {\text{for}}\quad m = 1,2,3, \ldots M, \hfill \\ \end{aligned}$$

where $S_{,k}^m$ represents the *k*-th column of $S^{m}$. We can cluster the nodes in each network from the first term in the objective function and obtain the alignment of clusters in different networks from the second term. The aligned clusters in all the views are obtained by solving optimization problem (11).

As shown below, the preceding optimisation problem is relaxed to a multiview spectral clustering optimization problem as follows:

12 $$\begin{aligned} \min _{X_{m}\in R^{N\times K}} \ Tr\left( X^{T} L X \right) \text { s.t. }X^{T}X=I, \end{aligned}$$

where

13 $$\begin{aligned} L= \begin{pmatrix} L_{1} &{} 0 &{} \cdots &{} 0 \\ 0&{} L_{2}&{} \cdots &{} 0 \\ \vdots &{} \vdots &{} \ddots &{} \vdots \\ 0&{} 0 &{} \cdots &{} L_{M} \end{pmatrix} -\beta \begin{pmatrix} 0 &{} I_{n} &{} \cdots &{} I_{n} \\ I_{n}&{} 0 &{} \cdots &{} I_{n} \\ \vdots &{}\vdots &{} \ddots &{} \vdots \\ I_{n}&{} I_{n} &{} \cdots &{} 0 \end{pmatrix} \, \ X= \begin{pmatrix} X_{1}\\ X_{2}\\ \vdots \\ X_{M} \end{pmatrix}. \end{aligned}$$

To relax both constraints in optimization problem (11), we transform the constraints for each network into a single equation with $X_{m}=\dfrac{S^m}{||S^m||_{2}}$. In the first constraint in optimization problem (11), the variables $S^m$ are relaxed from binary values to real values. To combine the information of all networks, we relaxed the second constraint to $X^{T}X=I_{K}$. However, this relaxation will result in the loss of information in the network [23]. To preserve information, we solve the following optimization problem:

14 $$\begin{aligned} \min _{X_{m}\in R^{N\times K}} \ Tr\left( X^{T} L X \right) \text { s.t. } X_{ m}^T X_{m}=I_{K}, \ \ m = 1;2;\dots ; M. \end{aligned}$$

Let $X_{m}=\dfrac{S^m}{||S^m||_{2}}$. Then, the constraint $X_{ m}^T X_{m}=I_{K}$ is direct relaxation of $\sum _{k=1}^{K} S_{,k}^m =1$, and it retains more information than in the optimization problem (11).

The problem (14) is an optimization problem with orthogonality constraints. The orthogonality constraints can be expressed as a Stiefel manifold $St_{n,p}$, which $X\in St_{n,p} =\left\{ {X \in R^{n\times p} | X^T X=I_{p}} \right\}$. Thus, problem (14) can be written as

15 $$\begin{aligned} \min _{{\left\{ X_{m}\right\} }_{m=1}^M\in St_{K,N}} \ \ Tr\left( X^{T} L X \right) , \end{aligned}$$

which is an optimization problem defined in manifold.

#### Solving the optimization problem

In this step, we solve optimization problem (15) by using the penalty model, i.e. the first-order algorithm from [33]. This algorithm iteratively implements steps.

*Step 1* As mentioned earlier, Xiao et al. [33] demonstrated that the original problem (1) and the penalty model 3 are equivalent. We begin by computing the gradient of the augmented Lagrangian [36] to approximate problem (15). The gradient of the augmented Lagrangian function with respect to *X* is formulated as

16 $$\begin{aligned} D_{m}= \nabla _{X}\varphi \left( X_{m},\delta \right) = \nabla f\left( X_{m}\right) + X_{m} \delta \left( X_{m}\right) +\beta X_{m} \left( X_{m}^{T}X_{m}-I_{p}\right) , \end{aligned}$$

where $D_{m}= \nabla _{X}\varphi \left( X_{m},\delta \right)$ denotes the gradient of the augmented Lagrangian, and $\nabla f\left( X_{m}\right)$ represents the gradient of the objective function 4. We computed the gradient of the objective function as

17 $$\begin{aligned} G=\nabla _{X} f\left( X\right) =\nabla _{X} Tr\left( X^{T}LX\right) =LX, \end{aligned}$$

and $\delta \left( X \right)$ is the Lagrangian multiplier. The PLAM method in [36] is shown as the closed-form expression of the Lagrangian multipliers $\delta \left( X\right)$, which is defined as

18 $$\begin{aligned} \delta \left( X\right) = \psi \left( \nabla f\left( X\right) ^{T} X\right) , \end{aligned}$$

where the operator $\psi$ is defined from Eq. (2) as follows,

19 $$\begin{aligned} \psi \left( X\right) =\dfrac{X^{T}+X}{2}. \end{aligned}$$

From Eqs. (18) and (19), we compute the Lagrangian multiplier to have the following form:

20 $$\begin{aligned} \delta \left( X\right) = \psi \left( \nabla Tr\left( X^{T}LX\right) ^{T} X\right) = \psi \left( \left( LX\right) ^{T} X\right) =\dfrac{X^{T}\left( L^{T}+L\right) X}{2}. \end{aligned}$$

We use Eq. (16) to calculate the gradient of the augmented Lagrangian.

*Step 2* We perform the following iterations in this step. We compute ${\tilde{X}}_{m+1}=X_{m}-\mu _{m}D_{m}$, and then we check if $\left\| X_{m+1} \right\| _{F}$ is greater than the parameter *r*. If yes, then

21 $$\begin{aligned} X_{m+1}=\frac{r}{\left\| {\tilde{X}}_{m+1} \right\| _{F}} {\tilde{X}}_{m+1}. \end{aligned}$$

If $\left\| X_{m+1} \right\| _{F}$ is not greater than *r*, then ${X}_{m+1}={\tilde{X}}_{m+1}$, where $\mu _{m}$ is the stepsize, and *r* is the radius. We explain how we selected the two parameters in the “Results and discussion” section. We repeat the processing of the aforementioned steps until convergence. Finally, we obtain the values of *X*.

#### k-means

We begin by setting *X* as $N\times M$ points in $R^{K}$, and then use *k*-means clustering to obtain the clusters. The output is the class label of the patients (nodes) in each network. We call our method multiview clustering by using the penalty model (MVCPM).

### Algorithm

In this subsection, we present the algorithm framework of the proposed method as shown in Algorithm 1. For convenience, we call it MVCPM.
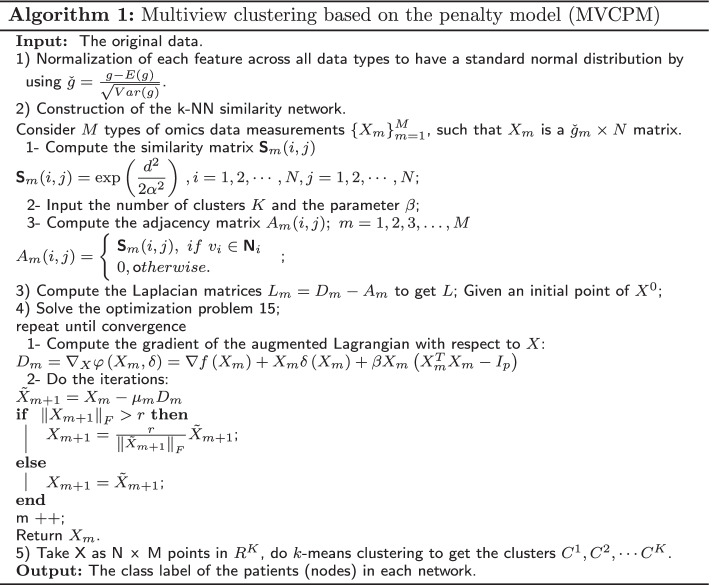


### Example for the proposed method

**Fig. 1:**
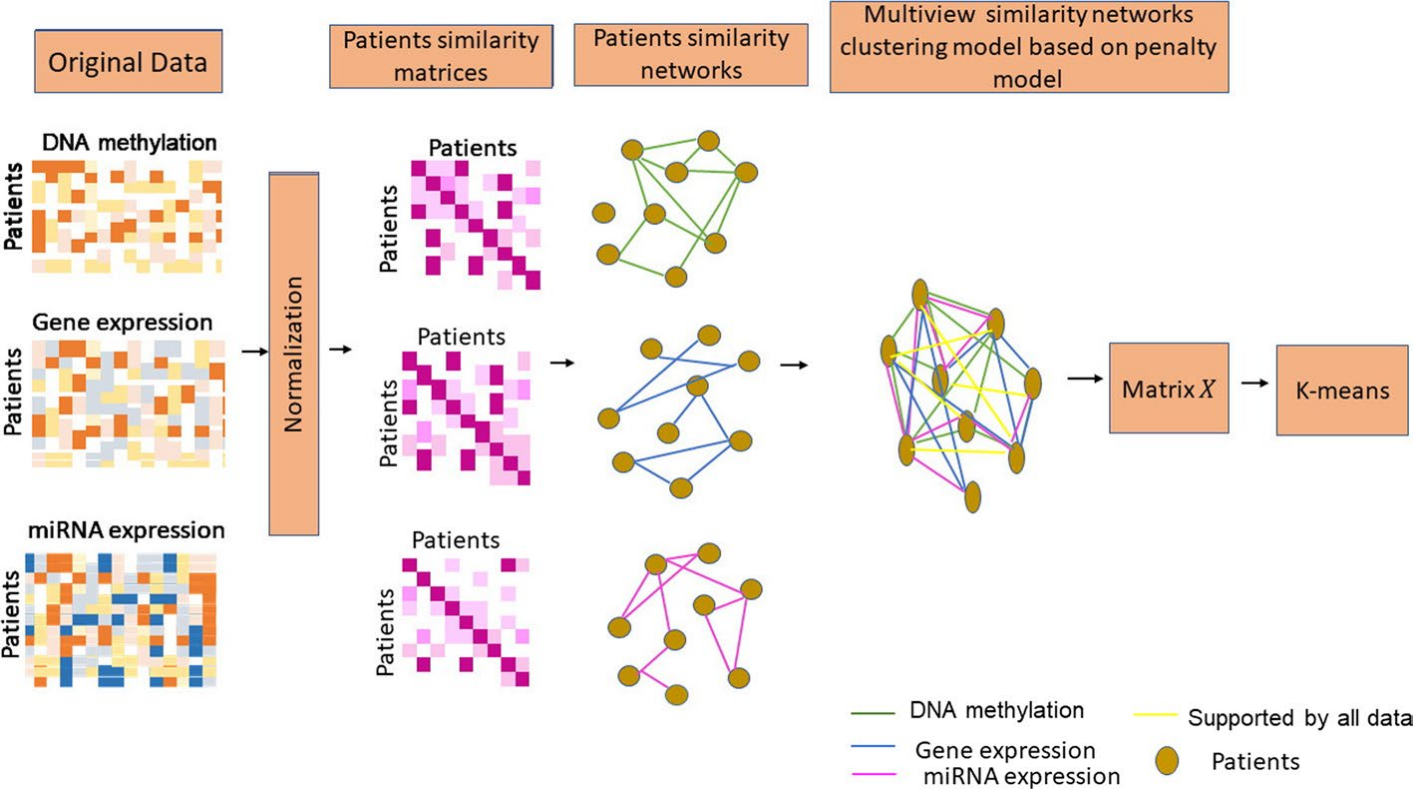
Illustrative example of method steps

Figure 1 presents an illustrative example of our method’s steps.

## Results and discussion

We investigate the performance of our method (i.e. MVCPM) in this section. The performance of the proposed method is compared with several recently developed methods by using synthetic and real data. The comparison methods are the affinity aggregation for spectral clustering (AASC) algorithm [34], the multiview clustering based on manifold optimisation (MVCMO) method [23] and the multiview spectral clustering (MVSC) method [21].

### Comparison using synthetic data

We begin by simulating network structures because all the methods mentioned earlier have been proposed using network tools. The networks are generated via the stochastic block model [39]. We begin by simulating network structures because all the methods mentioned earlier have been proposed using network tools. The networks are generated via the stochastic block model [39]. Assume that *M* views are present, and *N* is the number of nodes in each view. First, the number of *N* nodes is assigned to *K*. Then, the connections within and between different clusters are generated using a given probability matrix, in which diagonal entries specify connection probabilities within clusters. The other entries specify connection probabilities between corresponding clusters. To obtain the results when between cluster connections are changed, we use the following four connection probability matrices, with the within cluster connection probability being larger than that between clusters:

22 $$\begin{aligned} \begin{aligned} P_{1}&=\frac{1}{n} \begin{pmatrix} 16 &{} 0 &{} 0 \\ 0 &{} 18 &{} 0 \\ 0&{} 0 &{} 17 \end{pmatrix} \, \ \ P_{2}=\frac{1}{n} \begin{pmatrix} 16 &{} 0.4 &{} 0.6 \\ 0.4 &{} 18 &{} 0.55 \\ 0.6&{} 0.55 &{} 17 \end{pmatrix}\\ P_{3}&=\frac{1}{n} \begin{pmatrix} 16 &{} 0.8 &{} 1.2 \\ 0.8 &{} 18 &{} 1.1 \\ 1.2&{} 1.1 &{} 17 \end{pmatrix} \, \ \ P_{4}=\frac{1}{n} \begin{pmatrix} 16 &{} 1.2 &{} 1.8 \\ 1.2 &{} 18 &{} 1.65 \\ 1.8&{} 1.65 &{} 17 \end{pmatrix}. \end{aligned} \end{aligned}$$

The entries of main diagonal $\left( l,l\right)$ for each probability matrix represent the connection probabilities within cluster *l*, whilst the entries $\left( l,s\right) ;l\ne s$ represent the connection probabilities between the cluster *l* and cluster *s*.

To evaluate its performance, the proposed method is compared with existing methods in the literature under different scenarios. Firstly, the performance of the numbers of *M* views and *N* nodes is analysed when setting the same cluster size. For example, we set the cluster size as $\left( 50,50,50\right) N = 150$ and $M = 3$ to test the performance of the proposed method and as $\left( 200,200,200\right)$, $N=600$ and $M=5$ to emphasise the superiority of our presented method. Meanwhile, performance is analysed under different cluster sizes, i.e. cluster size is set as $\left( 50,50,50\right) ,\left( 30,90,30\right) ,\left( 40,60,50\right)$ with $N = 150$, $M = 3$, and $\left( 200,150,250\right) ,\left( 230,270,100\right) ,\left( 180,160,260\right) ,\left( 150,310,140\right) ,\left( 130,250,220\right)$ when $N = 600$ and $M = 5$.

To see the performance of all the methods, we repeat the experiments 50 times for each setting. Then, we calculate the identification accuracy of the clusters, wherein identification accuracy is represented by the Rand index, which is defined as

23 $$\begin{aligned} R=\dfrac{TP + TN }{TP + TN + FP + FN }, \end{aligned}$$

where *TP*, *TN*, *FP* and *FN* represent the numbers of true positive, true negative, false positive and false negative, respectively. *TP* is the number of nodes in clusters *i* and *j*, and the others can be defined similarly.

The results are presented in Tables 1, 2, 3 and 4. To see the results of the 50 times experiments, we list the mean Rand index and the standard deviation within brackets for each setting.

**Table 1:** Comparison between the performance of different methods, when the clusters size the same across all *M* views, $N = 150$, $M = 3$, and cluster size: $\left( 50,50,50\right) ,\left( 50,50,50\right) ,\left( 50,50,50\right)$

Method	$P_{1}$	$P_{2}$	$P_{3}$	$P_{4}$
MVCPM	1.00(0.00)	1.00(0.00)	1.00(0.00)	1.00(0.00)
MVSC	1.00(0.00)	1.00(0.00)	1.00(0.00)	0.99(0.01)
MVCMO	1.00(0.00)	1.00(0.00)	1.00(0.00)	0.99(0.01)
AASC	1.00(0.00)	1.00(0.00)	1.00(0.00)	1.00(0.00)

**Table 2:** Comparison between the performance of different methods, when the clusters size is different across all *M* views, $M=3$, $N=150$, and cluster size: $\left( 50,50,50\right)$, $\left( 30,90,30\right)$, $\left( 40,60,50\right)$

Method	$P_{1}$	$P_{2}$	$P_{3}$	$P_{4}$
MVCPM	1.00(0.00)	1.00(0.01)	0.97(0.00)	0.97(0.01)
MVSC	0.99(0.01)	0.98(0.01)	0.94(0.12)	0.89(0.16)
MVCMO	0.99(0.01)	0.99(0.01)	0.97(0.07)	0.97(0.02)
AASC	0.73(0.00)	0.73(0.00)	0.73(0.01)	0.73(0.01)

**Table 3:** Comparison between the performance of different methods. When the size of the cluster is the same across all *M* views, $M=5$, $N=600$, and cluster size: $\left( 200,200,200\right)$

Method	$P_{1}$	$P_{2}$	$P_{3}$	$P_{4}$
MVCPM	1.00(0.00)	1.00(0.00)	1.00(0.00)	1.00(0.00)
MVSC	1.00(0.00)	1.00(0.00)	1.00(0.00)	1.00(0.00)
MVCMO	1.00(0.00)	1.00(0.00)	1.00(0.00)	1.00(0.00)
AASC	1.00(0.00)	1.00(0.00)	1.00(0.00)	1.00(0.00)

**Table 4:** Comparison between the performance of different methods. When the size of the cluster is different across all *M* views, $M=5$,$N=600$, and cluster size:$\left( 200,150,250\right)$,$\left( 230,270,100\right)$, $\left( 180,160,260\right)$, $\left( 150,310,140\right)$, $\left( 130,250,220\right)$

Method	$P_{1}$	$P_{2}$	$P_{3}$	$P_{4}$
MVCPM	0.92(0.01)	0.93(0.00)	0.93(0.01)	0.89(0.02)
MVSC	0.91(0.01)	0.90(0.02)	0.77(0.06)	0.76(0.04)
MVCMO	0.90(0.01)	0.91(0.00)	0.90(0.02)	0.78(0.03)
AASC	0.56(0.56)	0.56(0.56)	0.56(0.56)	0.56(0.56)

As shown in Tables 1 and 3, when cluster sizes are the same across all *M* views, all four methods can cluster the nodes with a mean Rand index close to 1 and a lower standard deviation. Thus, all the methods exhibit comparable performance and demonstrate the significance of data integration, with AASC and our method performing the best with a ratio of 0.01.

Tables 2 and 4 present the results of the four methods when cluster sizes are different. Our method outperforms all the other methods. The primary reason is solving optimization problem 15. Most of the other methods formulate the optimization problem under the assumption that cluster sizes are the same across different networks. Then, they focus on finding the common features of the cluster to perform clustering. In AASC, for example, the *M* vectors that minimize the ratio cut of the weighted combination of all the networks are computed. By clustering these *M* vectors, the common cluster for all the considered networks can be obtained. In Tables 2 and 4, the mean Rand index of the AASC method is lower than those of the other methods. Consequently, all the methods can detect cluster differences between different views, whereas the AASC method regards them as the same. Thus, MVCPM, MVSC and MVCMO exhibit better performance than the AASC method. The MVCPM method outperforms MVSC and MVCMO, which has the highest mean Rand index. The MVCPM method performs more consistently when networks are fewer, resulting in a considerably lower standard deviation of the Rand index.

#### Selection of the parameters of the proposed method

In this section, we describe the method for choosing the values of the penalty parameter $\beta$, the stepsize $\mu _{k}$, and the radius *r*.

Penalty parameter $\beta$. In Theorem 4.1 [33], $\beta$ should be sufficiently large to guarantee convergence. Although, we can estimate a suitable $\beta$ that meets the requirements of previous theorems, such $\beta$ is too large to be practically helpful. In our method, we set $\beta$ to be less than $t=||f\left( X_{0} \right) ||$. In the selected penalty model, the compact convex set as the ball $B_{r}$ with radius $r> \sqrt{p}$.

*Stepsize* $\mu _{k}$ Practically, the upper bound of $\mu _{k}$ used in Theorem 4.1 is too restrictive. Therefore, we recommend using the alternating Barzilai–Borwein (ABB) step size [15] and the Barzilai–Borwein (BB) stepsize [40].

*ABB step size*

24 $$\begin{aligned} \mu _{m}^{ABB} = {\left\{ \begin{array}{ll} \mu _{m}^{BB1}, &{}for \ odd \ m\\ \mu _{m}^{BB2 }, &{}for \ even \ m. \end{array}\right. } \end{aligned}$$

*BB step size*

25 $$\begin{aligned} \mu _{m}^{BB1}=\frac{S_{m-1}^{T}S_{m-1}}{S_{m-1}^{T}Y_{m-1}} \ \, \ \mu _{m}^{BB1}=\frac{S_{m-1}^{T}Y_{m-1}}{Y_{m-1}^{T}Y_{m-1}}\, \end{aligned}$$

where $$S_{m-1}=X_{m}-X_{m-1}$$ and $$Y_{m-1}=D_{m}-D_{m-1}$$.

Moreover, we compare the penalty model for solving problem (15) with $n = 1000$, $p = 30$ and different pairs of $\beta$ and *r*: $\beta =10^{-6}t,10^{-5}t,10^{-4}t,10^{-3}t, 10^{-2}t,10^{-1}t,t,10t$ and $r= \sqrt{p},1.01\sqrt{p},1.04\sqrt{p},1.06\sqrt{p},1.3 \sqrt{p},1.8\sqrt{p},3\sqrt{p},7\sqrt{p},10\sqrt{p},\sqrt{p}$.

**Fig. 2:**
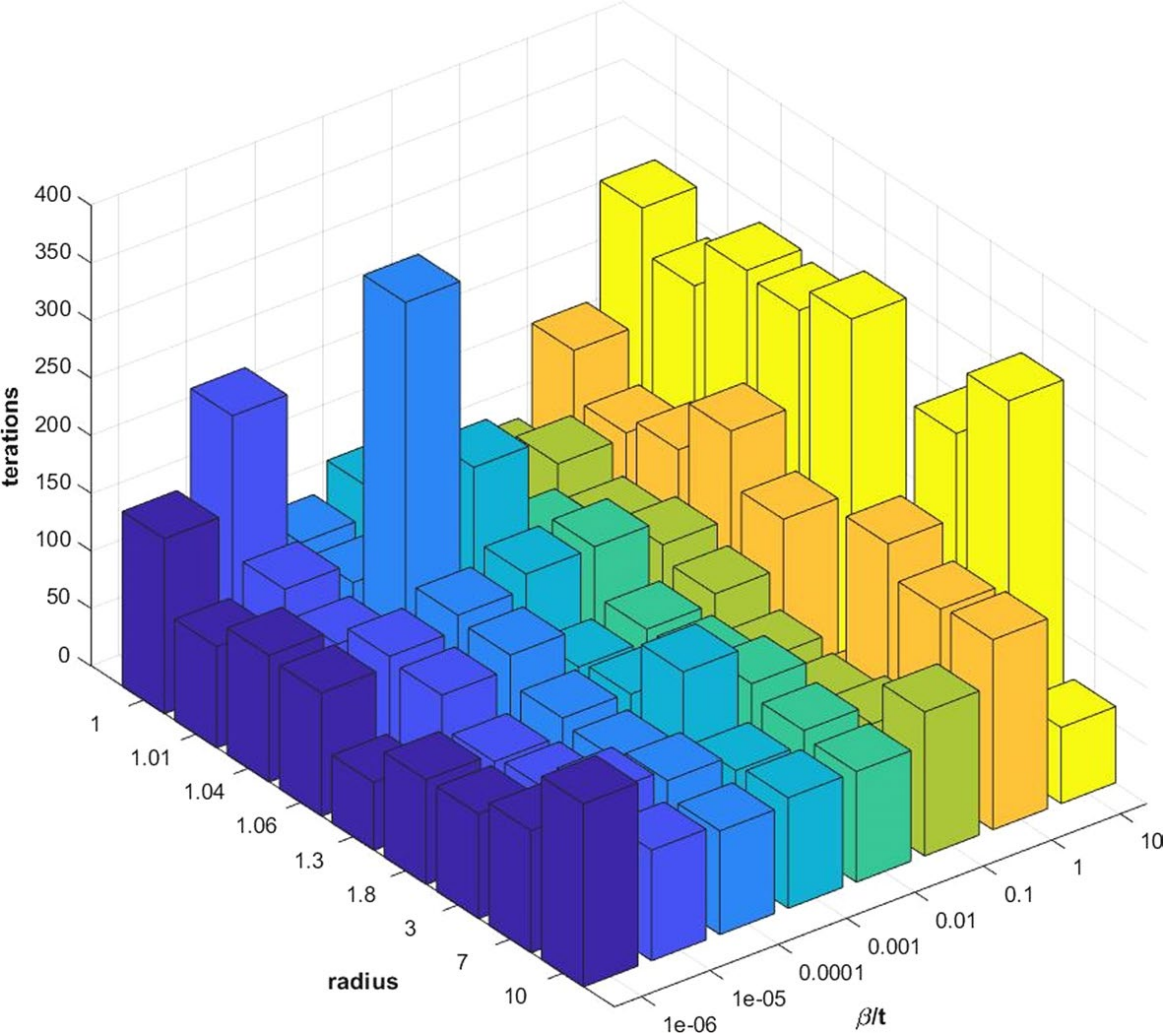
Show the results of the different settings for the parameters of the penalty model for solving the problem (15)

The results are presented in Fig. 2.

From Fig. 2, we can conclude that our algorithm is insensitive to the choice of *r* when $r \le 2$. Furthermore, a small $\beta$ can lead to a fast convergence rate. Consequently, we suggest choosing $\beta =0.01t$ whilst $r =1.04\sqrt{p}$.

To guarantee fair comparison over the literature, we set parameter $\beta =1$ to determine the performance of our method compared with those of other methods.

### Real data experiments

In this section, we use five real different types of cancer data to evaluate the performance of the proposed method and compare it with those of other methods. We select two commonly used measures, namely, silhouette score to measure clustering performance [41] and the P-value in the Cox log-rank test to evaluate the significance of difference between survival times [42].

#### Dataset

This study uses real multi-omics data generated by TCGA. We use the five data types of cancer processed in [16]. The data types are glioblastoma multiforme (GBM), lung squamous cell carcinoma (LSCC), kidney renal clear cell carcinoma (KRCCC) and colon adenocarcinoma (COAD). Each data type of cancer contains miRNA expression, DNA methylation and gene expression data type.

#### Comparison using silhouette score

We begin by constructing the *k*-NN similarity networks for each data type, choosing the five nearest neighbours and setting the number of clusters *K* as that in [16]. Then, we apply the proposed method to the *k*-NN similarity networks to obtain the final result.

We use the silhouette score to measure the clustering performance of our method (MVCPM) compared with the performance of other methods [41]. For the AASC method, we use the code developed in [34]. For the MVCPM and MVSC methods, we use the code generated in [23]. We compute the silhouette score of the common clusters and the average silhouette score of all data types to obtain the cluster assignment in each dataset and the common clusters across the three datasets. We also calculate the silhouette score of integrative clustering by using *k*-NN to assign patients who are previously clustered in different clusters into one cluster because cluster assignment in the three data types is not the same. We denote the silhouette score of the common clusters, the average silhouette score and the silhouette score for integrative clustering as (MVCPMcom, MVCMOcom, MVSCcom), (MVCPMavg, MVCMOavg, MVSCavg) and (MVCPMint, MVCMOint, MVSCint), respectively. The results are provided in Table 5.

**Table 5:** Comparisons all methods with real multi-omics data

Method	KRCCC	COAD	GBM	LSCC
MVCPMcom	0.5021	0.4169	0.4206	0.4236
MVCPMavg	0.4948	0.4100	0.4200	0.4437
MVCPMint	0.4904	0.3631	0.3870	0.3431
MVSCcom	0.3869	0.3853	0.2740	0.2345
MVSCavg	0.3782	0.3619	0.2395	0.2127
MVSCint	0.3786	0.2952	0.2136	0.1732
MVCMcom	0.4177	0.3958	0.2797	0.2475
MVCMOavg	0.3969	0.3725	0.2548	0.2296
MVCMOint	0.3581	0.2836	0.2063	0.1812
AASC	0.2815	0.2926	0.1944	0.1803

From Table 5, the common clusters identified using our method provide the highest silhouette score. The proposed method can capture common cluster structures efficiently in multiple views. The average silhouette scores are also higher than those using other methods in three of the four datasets. This result shows that MVCPM maintains the clustering results in each dataset. The cluster assignment in the three data types are not the same; thus, we use *k*-NN to assign patients that are originally clustered into different clusters into one cluster and compute silhouette scores as MVCPMint, MVCMOint, AASCint and MVSCint. In this case, the common silhouette score and average silhouette scores of all the methods are higher when compared with AASC, implying that all the methods, except for AASC, can identify the common clusters and capture the common cluster structures efficiently across multiview data. Moreover, all the methods have a high silhouette score for integrative clustering, except for AASC, indicating that all the methods can integrate clustering with good performance.

When we compare MVCPM, MVCMO and MVSC, MVCPM has the highest silhouette score for common clusters and the average silhouette score. MVCPM provides more detailed information within each data type, is better for integrating different types of omics data and simultaneously has consistent and differential cluster patterns. MVCPMcom and MVCPM avg are greater than MVCMO avg, MVCMOcom, MVSCavg and MVSCcom; therefore, our method has the highest silhouette score for integrative clustering. For example, MVCPMint in COAD is about 0.07 higher than MVCMOint, whilst MVCPMint is about 0.06 higher than MVSCint. Thus, MVCPM is the best method for integration the clustering.

#### Comparison by Cox survival P-values

In this subsection, we compare the performance of our proposed method with those of other methods by using the P-value in the Cox log-rank test to determine the significance of the difference in survival times [42]. The lowest P-values are used to determine the number of clusters for each cancer type in our proposed method. To ensure that the proposed method’s results are comparable with those of the other methods, we set the number of clusters for each cancer type for the other methods to be the same as the number of clusters for our proposed method. The results are provided in Table 6.

**Table 6:** Comparison of Cox survival p-values for all methods across all types of cancer

Cancer type	Our method MVCPM	MVCMO	MVSC	AASC
COAD (6cluters)	$3.7 \times 10^{-2}$	$6.5 \times 10^{-2}$	$3.4 \times 10^{-2}$	$13 \times 10^{-2}$
GBM (4cluters)	$1.2\times 10^{-4}$	$1.8 \times 10^{-2}$	$1.9 \times 10^{-3}$	$4 \times 10^{-3}$
LSCC (3cluters)	$8.6\times 10^{-3}$	$9 \times 10^{-3}$	$8.6\times 10^{-3}$	$8.4 \times 10^{-2}$
KRCCC (3cluters)	$38\times 10^{-2}$	$26\times 10^{-2}$	$38 \times 10^{-2}$	$41\times 10^{-2}$

**Fig. 3:**
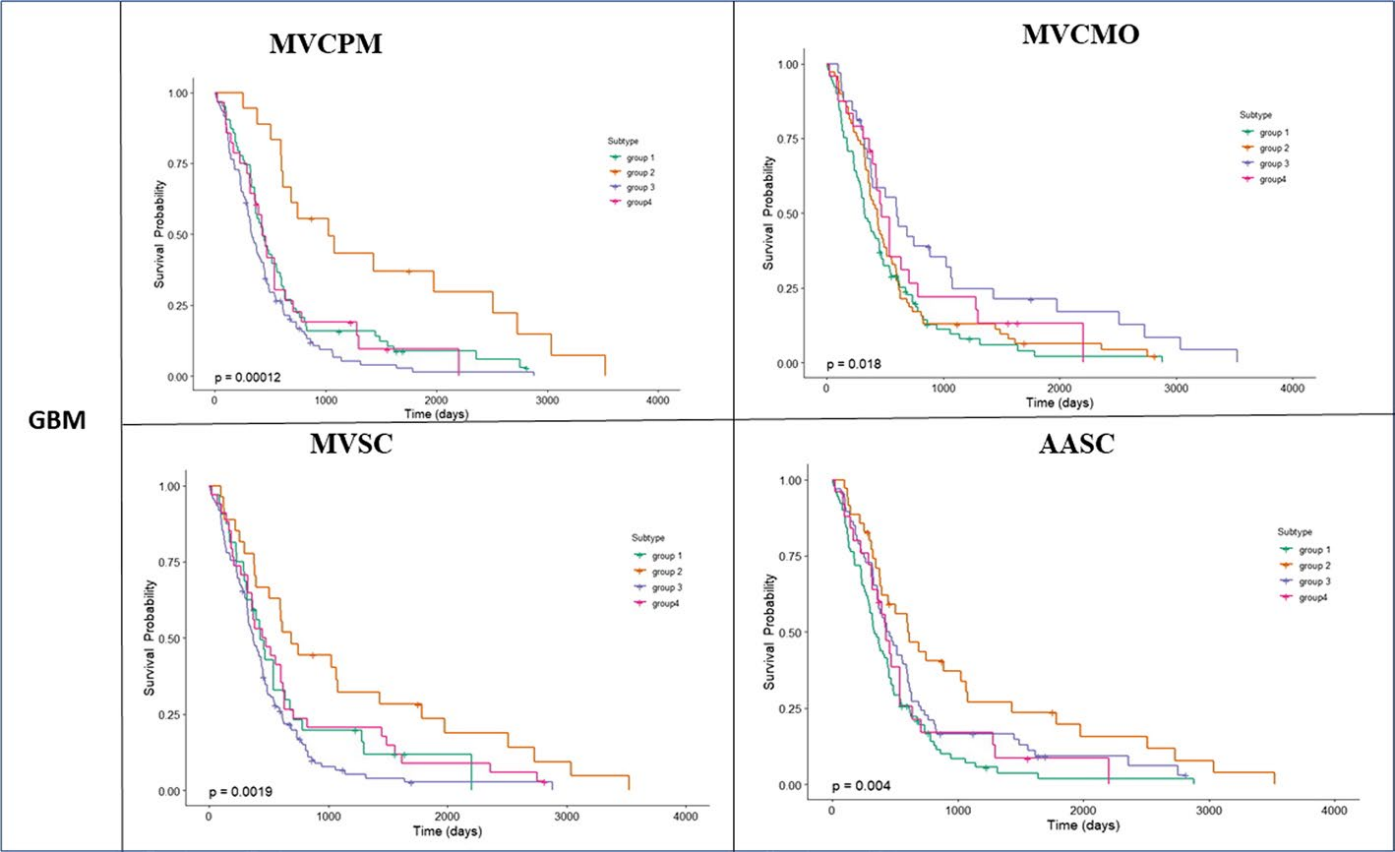
Kaplan-Meier survival curves of glioblastoma multiforme (GBM) of all methods (p-values are recorded in Table 6)

**Fig. 4:**
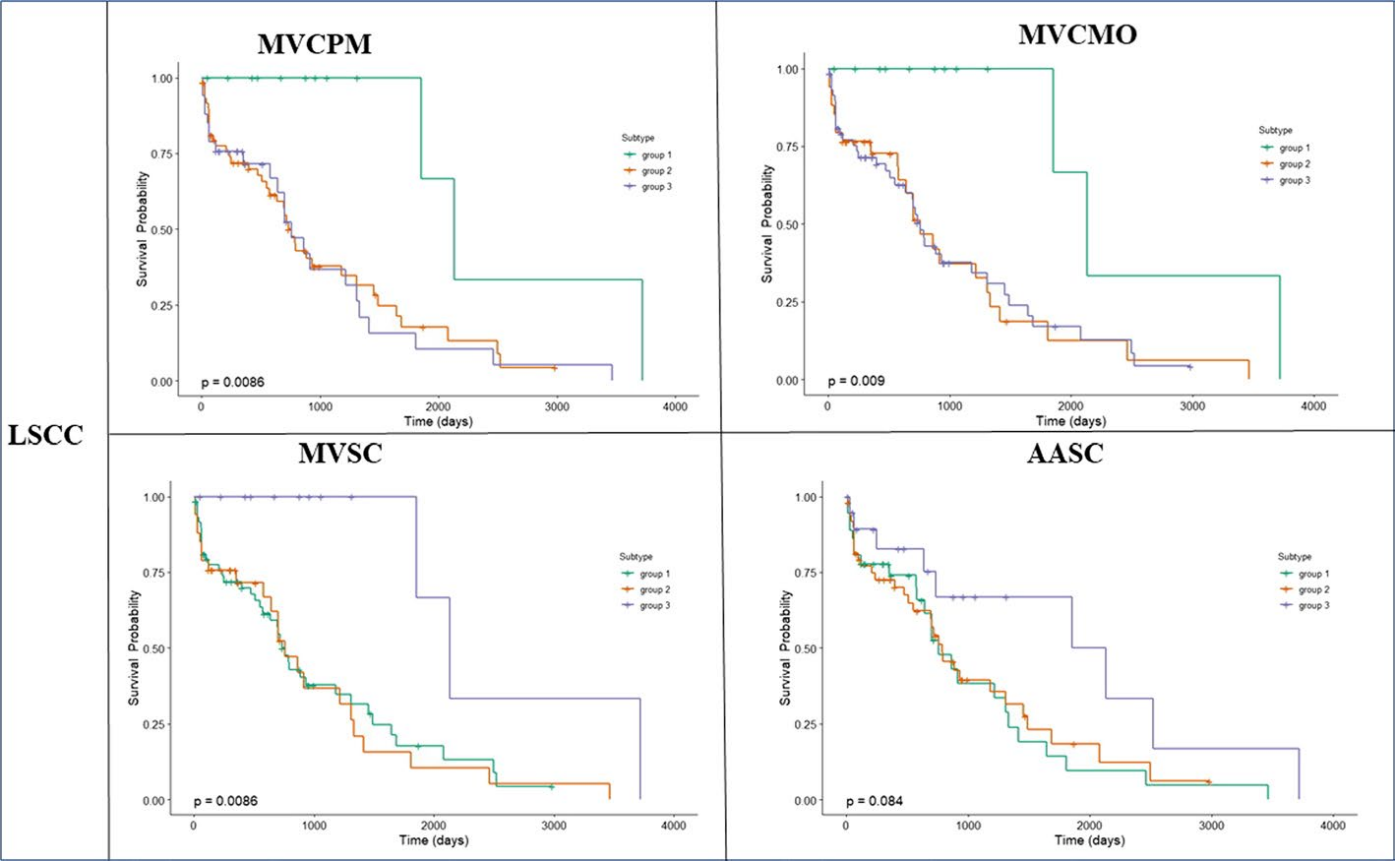
Kaplan-Meier survival curves of lung squamous cell carcinoma (LSCC) of all methods (p-values are recorded in Table 6)

**Fig. 5:**
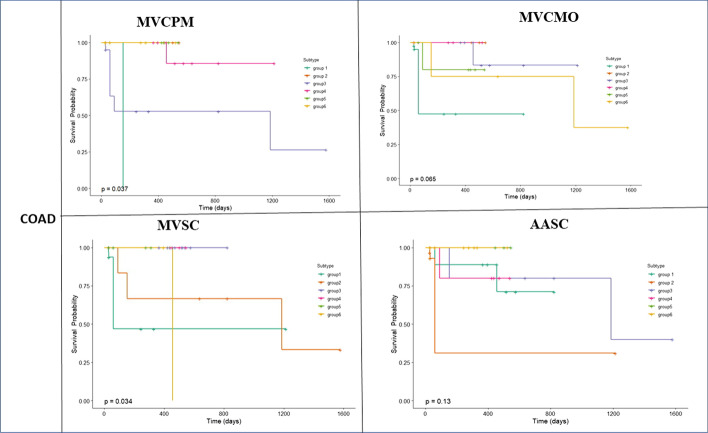
Kaplan-Meier survival curves of colon adenocarcinoma (COAD) of all methods (p-values are recorded in Table 6)

**Fig. 6:**
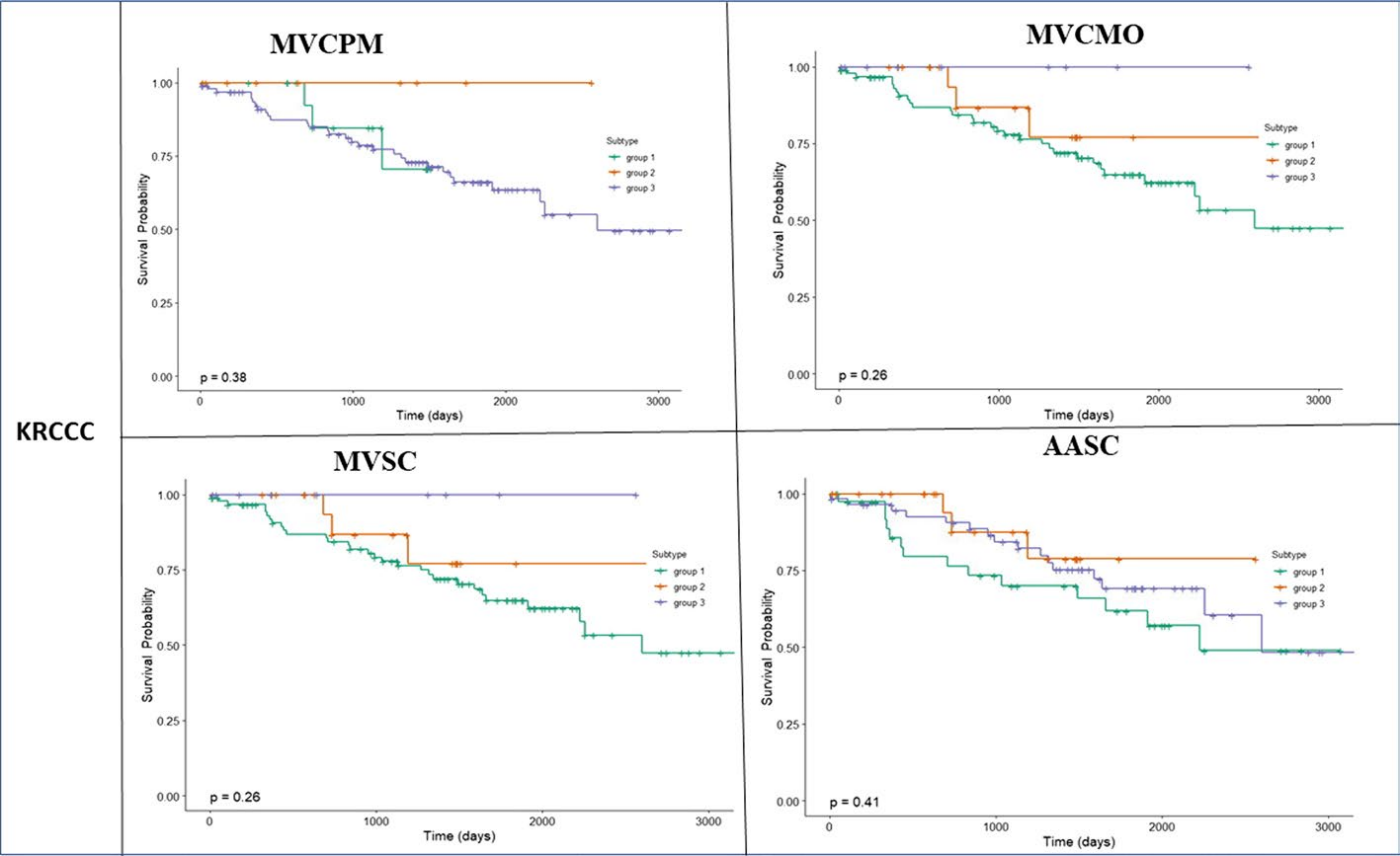
Kaplan-Meier survival curves of kidney renal clear cell carcinoma (KRCCC) of all methods (p-values are recorded in Table 6)

As indicated in Table 6, our method provides more significant differences in survival times across all types of cancer studied compared with the other methods. The P-value produced is comparable with the P-value produced by the other methods. Thus, the survival plots for all methods across COAD, GBM, KRCCC and LSCC cancers are presented in Figs. 3, 4, 5 and 6.

## Conclusion

This paper proposed a new method for multiview clustering of This study proposed a new method for the multiview clustering of multi-omics data based on a penalty model. Synthetic data and four different types of real multi-omics data were used to test the performance of the proposed method. We adopted the block model to generate these data in synthetic data and applied the proposed method and other methods. To ensure the performance of the proposed method, we compared it with some recently developed multiview clustering methods by using synthetic data. The results showed that when the underlying clusters were consistent, the proposed method exhibited competitive performance with the recently developed methods. However, the performance of the proposed method was better when the underlying clusters were differential.

For real multi-omics data, we downloaded the gene expression, miRNA expression and DNA methylation datasets for five different types of real multi-omics data. These are GBM, COAD, LSCC and KRCCC from TCGA. To test the clustering performance of all the methods, we used two commonly used measures: silhouette score to measure clustering performance and P-value in Cox log-rank test to evaluate the significance of difference between survival times. For the silhouette score, the proposed method obtained the highest silhouette score and provided more detailed information within each data type. In Cox survival P-values, the proposed method resulted in more significant differences in survival times across all studied data types.

Thus, the proposed method can be considered the best approach for integration and clustering. Although our method performed well in synthetic and real omics data analyses, some issues still need to be addressed. For example, we set parameter ${\beta =1}$ and the number of clusters to be three throughout our study to guarantee fair comparison with conventional methods. However, the number of clusters is not practically optional because it must be selected on the basis of the eigengap. Moreover, no guarantee theoretically exists to determine the optimal number of clusters. Consequently, developing an excellent statistical method for determining the optimal number of clusters in network-based clustering is still a worthwhile research.

## Data Availability

*Data sets* The datasets generated and/or analysed during the current study are available in the GitHub repository, https://github.com/hamas200/MVCPM.

## Abbreviations

MVCPM: Our method
AASC: Affinity aggregation for spectral clustering algorithm
MVCMO: Multiview clustering based on manifold optimization method
MVSC: Multiview spectral clustering method
GBM: Glioblastoma multiforme
LSCC: Lung squamous cell carcinoma
KRCCC: Kidney renal clear cell carcinoma
COAD: Colon adenocarcinoma

## Code availability

Matlab code for our method from (https://github.com/hamas200/MVCPM.) and Rstudio for analysis the p-value for all method described in this paper. The comparison methods code downloaded from http://homepage.fudan.edu.cn/sqzhang/files/2018/12/MVCMOcode.zip.
